# Purification and Characterisation of Immunoglobulins from the Australian Black Flying Fox (*Pteropus alecto*) Using Anti-Fab Affinity Chromatography Reveals the Low Abundance of IgA

**DOI:** 10.1371/journal.pone.0052930

**Published:** 2013-01-07

**Authors:** James W. Wynne, Antonio Di Rubbo, Brian J. Shiell, Gary Beddome, Christopher Cowled, Grantley R. Peck, Jing Huang, Samantha L. Grimley, Michelle L. Baker, Wojtek P. Michalski

**Affiliations:** 1 Australian Animal Health Laboratory, CSIRO Animal, Food and Health Sciences, Geelong, Victoria, Australia; 2 School of Life Science, East China Normal University, Shanghai, China; National Institute on Aging, United States of America

## Abstract

There is now an overwhelming body of evidence that implicates bats in the dissemination of a long list of emerging and re-emerging viral agents, often causing illnesses or death in both animals and humans. Despite this, there is a paucity of information regarding the immunological mechanisms by which bats coexist with highly pathogenic viruses. Immunoglobulins are major components of the adaptive immune system. Early studies found bats may have quantitatively lower antibody responses to model antigens compared to conventional laboratory animals. To further understand the antibody response of bats, the present study purified and characterised the major immunoglobulin classes from healthy black flying foxes, *Pteropus alecto*. We employed a novel strategy, where IgG was initially purified and used to generate anti-Fab specific antibodies. Immobilised anti-Fab specific antibodies were then used to capture other immunoglobulins from IgG depleted serum. While high quantities of IgM were successfully isolated from serum, IgA was not. Only trace quantities of IgA were detected in the serum by mass spectrometry. Immobilised ligands specific to IgA (Jacalin, Peptide M and staphylococcal superantigen-like protein) also failed to capture *P. alecto* IgA from serum. IgM was the second most abundant serum antibody after IgG. A survey of mucosal secretions found IgG was the dominant antibody class rather than IgA. Our study demonstrates healthy *P. alecto* bats have markedly less serum IgA than expected. Higher quantities of IgG in mucosal secretions may be compensation for this low abundance or lack of IgA. Knowledge and reagents developed within this study can be used in the future to examine class-specific antibody response within this important viral host.

## Introduction

Bats represent approximately one fifth of the world's mammalian species and are among the most diverse and geographically dispersed mammals. Frugivorous and nectivorous pteropid bats (family *Pteropodidae*, suborder *Megachiroptera*) constitute approximately 170 species and are known colloquially as fruit bats or flying foxes. Their capacity for long-distance dispersal through flight has undoubtedly contributed to their widespread distribution throughout tropical and sub-tropical Asia and Australia, and on islands of the Indian and western Pacific Oceans. The black flying fox, *Pteropus alecto*, is distributed mainly across the northern and eastern coasts of Australia [1]. Since 1994, this species has gained particular attention after it was identified as the natural host of Hendra virus (HeV) [2], [3].

A body of evidence exists implicating bats as a major source of zoonotic viruses [4]–[6]. Bats have been shown to harbour and disseminate highly pathogenic viruses including Ebola, SARS-like coronavirus and the henipaviruses (HeV and Nipah) [4]. Spillover events from bats to other species – including humans – have increased. In 1998, a spillover event of Nipah virus from pteropid bats to farmed pigs caused a major disease outbreak in Malaysia. More than 1 million pigs were culled, and of the 265 reported human cases, 105 were fatal [7], [8]. While the incidence of HeV in Australia has been more sporadic, an increase in spillover events from pteropid bats to horses has occurred. Between 1994 and 2010, 14 HeV outbreaks occurred in Australia involving 48 confirmed equine cases (75% fatality rate) and seven human cases (60% fatality rate). In contrast, between June 2011 and January 2012, 19 HeV outbreaks occurred [9]. The increasing incidence of HeV, combined with its high fatality rate, means HeV has the potential to cause further significant disease outbreaks in Australia and abroad. Recently, a novel paramyxovirus that is closely related to the henipaviruses, named Cedar virus, was isolated from fruit bats in Australia [10]. However unlike HeV, Cedar virus caused no clinical disease in experimentally infected ferrets and guinea pigs [10]. Evidence of henipavirus infection in multiple bat species has also been identified in continental Africa [11].

Despite the risk of zoonotic diseases originating in bats, little is known regarding the bat immune system and very few diagnostic reagents are commercially available. Despite their ability to carry highly pathogenic viruses such as Hendra, Nipah and Ebola, bats appear asymptomatic and rarely show signs of disease following both experimental and natural infection with these agents [12]–[16]. The notable exceptions are rabies-like viruses, which are capable of causing clinical disease in bats and humans [17], [18]. Anecdotal observations raise the possibility that the bat immune system may have atypical and possibly unique characteristics that permit asymptomatic virus persistence and spillover events.

Immunoglobulins, namely IgG, IgA and IgM, are important and diverse secretory components of the mammalian immune system. Antibodies of the IgG class are the most abundant immunoglobulins in mammalian serum and consequently provide the majority of immunity against blood-borne infectious agents [19]. In contrast, IgA is most abundant in mucosal secretions including milk, tears, and secretions of the upper respiratory tract and digestive tract. The abundance of IgA in normal human serum is approximately one fifth of the IgG level and accounts for about 15–20% of total immunoglobulins in serum [20]. In humans, serum IgA is predominantly monomeric whereas IgA within mucosal secretions is found as a dimer complex. Polymerisation of the IgA (and IgM) is facilitated by the joining (J) chain which is expressed by immunocytes of various secretory tissues [21]. Polymeric IgA is secreted into the mucosa through its interaction with the polymeric Ig receptor (pIgR) which is expressed at the basolateral surface of secretory epithelial cells. The IgA/pIgR complex is transported via transcytosis to the cell surface where it is ultimately secreted in to the lumen [22]. Mammalian IgA plays a crucial role in mucosal immunity while in serum it interacts with the IgA Fc receptor (FcαR) to control the inflammatory response [20]. Indeed, in the absence of antigen, IgA down-regulates IgG mediated phagocytosis, chemotaxis, cytokine release and oxidative burst activity [23]–[26]. IgM is the first antibody produced following microbial invasion and exists predominantly as either a monomeric antigen receptor on B cells or as a soluble pentamer. IgM can also exist as tetramer and dimers, however these forms are significantly less abundant. In healthy humans serum IgM is slightly less abundant compared to serum IgA. Variation in the nature of *N*-linked glycosylation provides further diversity to immunoglobulins. These glycans play a vital role in maintaining structural integrity and in some cases act as ligands for immunologically important serum lectins such as mannan-binding lectin [27].

Few studies have examined the immunoglobulin system of bats. Recently Baker *et al.* [28] sequenced mRNA encoding the heavy chains of IgG, IgM and IgA of *P. alecto*, revealing a diverse repertoire of variable heavy chain transcripts. Transcripts resembling IgE have also been indentified in different bat species, while IgD appears specific to only insectivorous bats [29]. Wild-caught bats are certainly capable of generating neutralising antibodies to viruses such as Hendra, Ebola and SARS-like coronavirus [2], [30], [31], but early studies have demonstrated pronounced differences in both the magnitude and duration of antibody responses in bats compared to other mammals [32]–[34]. The large frugivorous bat, *P. giganteus*, demonstrated a delayed primary antibody response following immunization with sheep red blood cells [32]. Similarly, the magnitude and duration of the neutralising antibody response of the Big Brown Bat (*Eptesicus fuscus*) to the model antigen PhiX174 bacteriophage was lower than that of rabbits and guinea pigs [33]. The likelihood that bats have an atypical immunoglobulin response compared to other animals requires further attention. Specifically, the abundance, tissue distribution and post-translational modification of the different immunoglobulin classes must be examined.

Given the importance of bats as viral reservoirs, understanding the bat immune system may facilitate development of methods to mitigate spillover events in the future. As a first step, this study aimed to purify and characterise the major immunoglobulin classes from healthy *P. alecto* biological specimens. Considering that in other mammalian species, immunoglobulins IgG, IgM and IgA are present in relatively high abundance in serum and tissues, we anticipated that bats would possess a similar immunoglobulin profile. However, while IgG and IgM appeared abundant in *P. alecto* serum, IgA was not. IgA was detected in the mucosal secretions of the small and large intestine lavages, milk and tears. Diverse isoforms of IgG and IgM, suggestive of multiple subclasses, were identified. Reagents developed within this study will aid future studies of this unique immunoglobulin repertoire, particularly in response to viral infection.

## Materials and Methods

### Animals and sample preparation

All animal experimentation and sample collection was conducted following guidelines approved by the AAHL Animal Ethics Committee (permit no. 1302). *P. alecto* bats were captured in southern Queensland, Australia as described previously [35] and transported live by air to the CSIRO Australian Animal Health Laboratory (AAHL). The animals were bled for serum and plasma and then euthanized for dissection of tissues. Tissues were stored at −80°C in RNA*later* (Ambion) for RNA analysis or snap frozen in liquid nitrogen for downstream mass spectrometry (MS) analysis. Lungs, small and large intestines were washed with 15–20 ml of cold phosphate buffered saline (PBS). Washes (lavages) and tissues were stored at −80°C. Faeces samples were collected from bat cages within 1–2 hours of being excreted and immediately resuspended in PBS containing protease inhibitors as previously described [36]. Where indicated, serum was extracted from plasma according to the protocol described by Salvador-Morales *et al.* [37]. All chemical reagents were of the highest analytical grade available from Merck, unless otherwise specified.

### Nomenclature

Throughout this paper, whole immunoglobulin proteins are designated as either IgG, IgA and IgM. The heavy chain protein components are designated IgG_H_, IgA_H_ and IgM_H_, respectively. Constant regions for IgG_H_, IgA_H_ and IgM_H_ are designated Cγ, Cα and Cμ, respectively. Immunoglobulin genes are designated *IGHG*, *IGHA*, *IGHM*, *IGJ* and *PIGR* for IgG_H_, IgA_H_, IgM_H_,the joining chain and polymeric immunoglobulin receptor, respectively.

### Purification strategy

**Figure 1:**
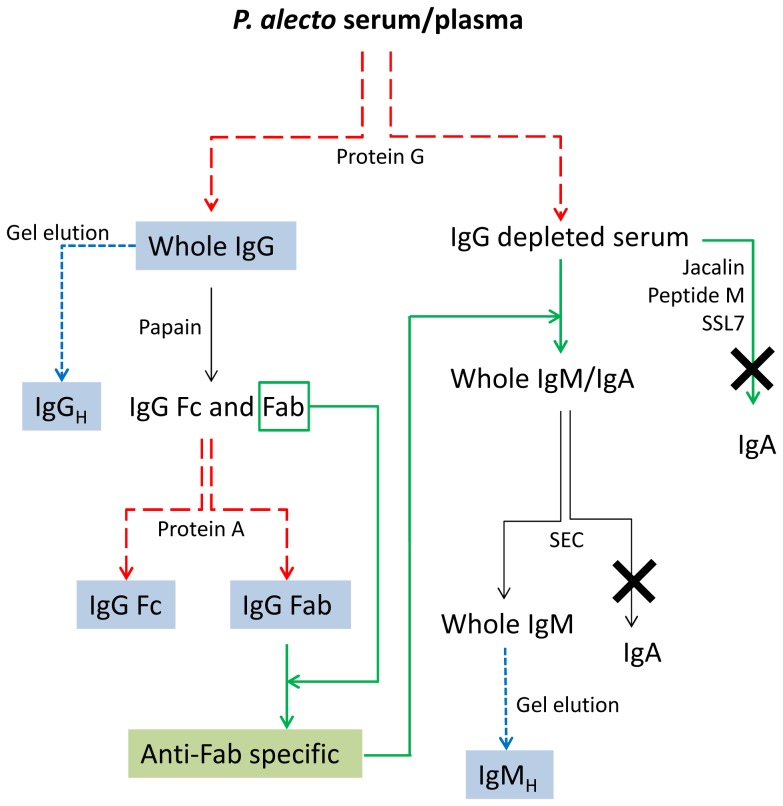
Purification strategy of *P. alecto* IgG, IgM and IgA. *Light blue* boxes represent those components which polyclonal antiserum was raised against. The *green* box represents *P. alecto* anti-Fab-specific antibody. *Red dashed* lines represent Protein G or Protein A affinity chromatography, *blue dashed* lines represent heavy chain gel elution, *solid green* lines represent specific affinity chromatography. Crosses represent unsuccessful purification of IgA. Abbreviations: SEC; size exclusion chromatography.

The purification strategy employed in this study exploited the molecular characteristics of isotypes of mammalian immunoglobulins with the assumption that bat immunoglobulins would resemble those of other mammals. Serum and plasma samples from *P. alecto* bats were used as a source of immunoglobulins and human serum was used as a control. Immobilised Proteins A, G and L were used in this study to capture IgG from *P. alecto* serum. Fab fragments, derived by papain digestion of purified *P. alecto* IgG, were used to generate Fab-specific antibodies in rabbits. In turn, immobilised anti-Fab-specific antibodies were employed to capture remaining *P. alecto* immunoglobulins, from IgG-depleted bat serum (Fig. 1). The final separation of IgM from IgA was attempted by size exclusion chromatography (SEC) exploiting significant molecular mass difference between the two molecules or by reducing sodium dodecyl sulfate polyacrylamide gel electrophoresis (SDS-PAGE) separation of respective heavy chains. The identity of separated proteins was determined by comparing tandem mass spectrometric data with available protein sequence databases.

### Isolation of IgG from human, rabbit and *P. alecto* sera

Affinity chromatography purification of IgG from mammalian sera using immobilised Protein A, G or L was performed according to a method modified from that described by Bjorck and Kronvall [38]. Protein A and Protein G affinity columns (GE Healthcare) or Protein L affinity columns (Thermo) were connected to a 280 nm UV detector and a chart recorder. Serum samples were diluted 5-fold in binding buffer (20 mM phosphate buffer, pH 7.4) and loaded on to columns equilibrated with the binding buffer at a flow rate of 0.5 ml/min. Columns were washed with 10 volumes of binding buffer and IgG was eluted in elution buffer (100 mM glycine HCl, pH 2.7). Collected fractions were immediately pH neutralised with 1 M Tris-HCl, pH 9.0. Protein concentration was determined using the Micro BCA protein assay (Thermo) and protein samples were analysed by reducing SDS-PAGE.

### Digestion of purified *P. alecto* IgG with papain

Purified *P. alecto* and rabbit IgG samples (1 mg) were diluted with PBS and concentrated 5-fold to 200 µl using 4 ml Ultra-4 centrifugal filter devices with a 5 kDa molecular weight cut-off (MWCO) membrane (Amicon). The concentrate was diluted with 100 mM sodium acetate buffer, pH 5.8, containing 50 mM cysteine and 0.5 mM ethylenediaminetetraacetic acid (EDTA). Fifty microlitre aliquots were digested with increasing concentrations of papain (ranging from 0.01 to 0.05 mg/ml or 1∶100 to 1∶20 enzyme to substrate ratios, respectively) over different incubation periods (6 to 22 h) at 37°C. The reactions were stopped by addition of 30 mM iodoacetamide (Sigma) and incubated at room temperature for 30 min. The extent of the digestion was assessed by reducing SDS-PAGE. The optimal digestion conditions obtained for bat IgG (1∶20 enzyme to substrate ratio, 18 h) were used for digestion of 20 mg of IgG in scaled-up experiments.

### Separation of *P. alecto* IgG Fab fragment on immobilised-Protein A by affinity chromatography

Following large scale digestion, the papain digest was concentrated 5-fold to 200 µl using Ultra-4 centrifugal filter devices with a 5 kDa MWCO membrane and buffer exchanged with Protein G affinity chromatography binding buffer. This fraction contained IgG fragments (Fab and Fc) and was diluted 25-fold with the binding buffer prior to fractionation on an immobilised Protein A affinity column. *P. alecto* IgG Fc fragments bound to the column whilst Fab fragments were not retained by the column. The Fab and Fc fraction was tested for purity by reducing SDS-PAGE and used to immunise rabbits to generate Fab-specific antiserum. The IgG fraction of this rabbit antiserum was obtained by purification on an immobilised Protein A affinity column as described above.

### Coupling of Fab and rabbit anti-Fab antibodies to N-hydroxysuccinimide (NHS)-activated Sepharose

The purified *P. alecto* Fab fragments (5 mg) obtained by digestion of *P. alecto* IgG with papain were concentrated and buffer exchanged with 0.5 M NaCl in 0.2 M sodium bicarbonate buffer, pH 8.3, using Amicon Ultra-4 centrifugal filter devices. Coupling to a 1 ml HiTrap NHS-activated HP column (GE Healthcare) was accomplished according to the manufacturer's instructions. Rabbit anti-Fab antibodies were purified by affinity chromatography by applying the IgG fraction to the immobilised-Fab column using the procedure described for the purification of IgG. The purified antibodies, designated anti-Fab-specific antibodies (4 mg), were coupled to a HiTrap NHS activated HP column as described above.

### Purification of *P. alecto* IgM and IgA

IgG-depleted *P. alecto* serum and plasma samples were obtained by repeated (2–3 times) affinity chromatography on immobilised Protein G affinity columns. The flow through was assessed for the absence of IgG by reducing SDS-PAGE and western blot analysis with Protein G or whole *P. alecto* anti-IgG rabbit serum as detecting agents. IgG-depleted samples were fractionated by affinity chromatography on immobilised anti-Fab-specific antibodies adopting the same procedure as that described for immobilised Protein A and G except that the binding and washing buffer consisted of 0.3 M NaCl in 50 mM phosphate buffer, pH 7.4. All fractions were assayed for protein concentration and analysed by reducing SDS-PAGE.

Affinity chromatography media reported to specifically interact with mammalian IgA, including Jacalin [39], Peptide M [40] and staphylococcal superantigen-like protein 7 (SSL7) [41], were also used in attempts to purify *P. alecto* IgA from serum, plasma and tissue lavages. The affinity procedures were first tested on human serum and plasma samples and proved suitable for isolation of human IgA. The efficiency of purification steps was assessed by reducing SDS-PAGE and mass spectrometry.

### Protein elution from gels and preparation of rabbit antiserum

Affinity-purified IgG, Fab and Fc fragments from papain digests, and SEC-purified IgM fractions (250 µg) were separated by preparative reducing SDS-PAGE using large well 4–12% gradient Bis-Tris gels (Invitrogen, Carlsbad, CA, USA) under reducing conditions. The protein bands corresponding to heavy chain subunits of immunoglobulins were electroeluted from the gel using a Bio-Rad Mini Whole Gel Eluter according to the manufacturer's instructions with 0.01% SDS, 60 mM Tris, 40 mM N-cyclohexyl-3-aminopropanesulfonic acid (CAPS), pH 9.5, as eluting buffer or by passive elution overnight in PBS buffer containing 0.1% SDS [42]. The eluted fractions were analysed by reducing SDS-PAGE, pooled and used for production of specific antiserum in rabbits. Purified antigens (50 µg IgG; Fc fragment; Fab fragment; IgM_H_ and IgG_H_) were emulsified with CSIRO triple adjuvant (Montanide/Quil A/DEAE-dextran; [43]) to a final volume of 500 µl and injected intramuscularly at two sites at approximately 3 weekly intervals for 10 weeks.

### SDS-PAGE, 2-D electrophoresis (2-DE) and immunodetection

Protein fractions were analysed using reducing SDS-PAGE under reducing conditions in precast gels (4–12% Bis-Tris gels in a MOPS/SDS buffer or in 12% gels in Tris-glycine/SDS, Invitrogen). Protein bands were visualized after staining with either Coomassie Brilliant blue (CBB) or silver nitrate.

Serum samples (15 µg) were analysed by 2-DE as described previously [44]. Proteins were either visualized by silver nitrate or western transferred onto polyvinylidene fluoride (PVDF) membranes in 10 mM CAPS buffer, pH 11, containing 10% methanol for immunoblot analysis. Membranes were blocked with 5% skim milk overnight at 4°C, then probed with specific rabbit antiserum diluted 1∶10,000 or 1∶20,000 followed by goat anti-rabbit IgG horseradish peroxidase (HRP) conjugates diluted 1∶20,000 or 1∶40,000, respectively. Visualization of reactive protein bands was achieved by enhanced chemiluminescence (ECL, GE Healthcare) and exposure to X-ray film (Fuji).

### Glycan analysis

Samples of purified IgG and IgM from *P. alecto* and human sera were denatured with 0.25% Triton X-100 (ICN Biomedicals) and treated with *N*-glycosidase F (PNGaseF, Roche) or neuraminidase (from *Arthobacter ureafaciens*, Roche; relative rate of cleavage is α2→6>α2→3>α2→8) at 37°C overnight. Samples were separated by reducing SDS-PAGE and western transferred onto low fluorescence PVDF transfer membranes (Fluorotrans, Pall) as described above and then blocked overnight in 50 mM Tris-HCl, 150 mM NaCl and 0.25% Tween 20, pH 7.4 (TBS-T). After washing 3 times with TBS-T, membranes were incubated for 1 h with biotinylated lectins (Vector Labs Burlingame, CA, USA): *Datura stramonium* agglutinin (DSA; 1∶3,000 dilution), *Galanthus nivalis* agglutinin (GNA; 1∶3,000), *Maackia amurensis* agglutinin II (MAA II; 1∶1,000), *Sambucus nigra* agglutinin (SNA; 1∶30,000), peanut agglutinin (PNA; 1∶1,000) and *Ulex europaeus* agglutinin I (UEA I; 1∶1,000). After lectin incubation, the blots were washed three times in TBS-T for 10 min and then incubated for 1 h with Streptavidin-HRP conjugate (twice the dilution of lectin; Dako, NSW, Australia) in TBS-T followed by three 10 min washes, two in TBS-T then one in TBS. Visualization of reactive protein bands was achieved by 5 min incubation with enhanced chemiluminescence substrate (ECL Plus™) and scanning on a Typhoon FLA 9000 Gel Imaging Scanner (GE Healthcare).

### Chromatography

Preparative SEC was performed on a HRLC system (Bio-Rad) with UV monitoring of the eluate at 280 nm. The column used for separation was a Bio-Gel 40XL column (Bio-Rad) and the mobile phase consisted of 50 mM Na_2_PO_4_, 300 mM NaCl, pH 7.4. Eluted fractions were manually collected and subjected to analysis by reducing SDS-PAGE and mass spectrometry.

### Mass Spectrometry

Purified proteins were analysed by mass spectrometry of bands excised from CBB-stained bands from reducing SDS-PAGE gels and subjected to in-gel digestion with trypsin [45]. Resultant peptides were analysed by liquid chromatography tandem mass spectrometry (LC-MS/MS) using a Surveyor high-performance liquid chromatography (HPLC) system connected directly to the nano-spray ion source of an LCQ Classic quadrupole ion-trap mass spectrometer (Thermo) as described previously [46]. Identification of proteins was obtained using the TurboSequest program [45] (V3.2) (Thermo). MS/MS spectra of HPLC-separated peptides from the in-gel tryptic digests was matched with theoretical mass spectra produced by *in silico* tryptic digestion of protein sequences from the Ensembl *Pteropus vampyrus* protein database (http://asia.ensembl.org/info/data/ftp/index.html) and published sequences [15]. Search results were filtered by a single threshold where matches to peptides were reported only if the Sequest cross correlation factor (Xcorr) was >1.5 for a singly charged peptide ion, >2.0 for a doubly charged peptide ion or >2.5 for a triply charged peptide ion. Furthermore, a protein was reported as identified only if 2 or more different peptide matches were found for that protein.

### Quantitative real-time reverse-transcriptase PCR (qPCR)

Total RNA was extracted from lymph node, spleen, liver, lung, heart, kidney, small intestine, brain and salivary gland as previously described [47]. For each sample, 500 ng total RNA was used as template in a 20 µl cDNA synthesis reaction. Quantitative PCR primers were designed using Primer Express 3.0 (Applied Biosystems) with default parameter settings. *IGHG* primers: CCGGGAGCCGCATGTA (Fwd), TCCTTGGCCAGCTCATCTG (Rev), *IGHA* primers: CCCGGCTGAAGAGAAAAAGAC (Fwd), GGCACCGTGAGGGTAGGAT (Rev), *IGHM* primers: AGGAGCTGGCCTCGATCAC (Fwd), ACCGCACGAACACGTCTGA (Rev), *IGJ* primers: CCCACCTCACCAAGGAGAAC (Fwd), TGCAGGGTCACAATTTTTACAGA (Rev), *PIGR* primers: CTTGGCAACTCTTCGTCAATGA (Fwd), TCCTACCACACTCTTCACCACAGA (Rev), 18 s ribosomal RNA primers: CACGGCGACTACCATCGAA (Fwd), CGGCGACGACCCATTC (Rev). qPCR reactions were preformed in triplicate as described previously [47]. Copy numbers of target sequences were calculated using standard curves and normalised relative to 18 s ribosomal RNA.

## Results

### Purification of IgG and IgM

#### Purification of IgG and production of antiserum

**Figure 2:**
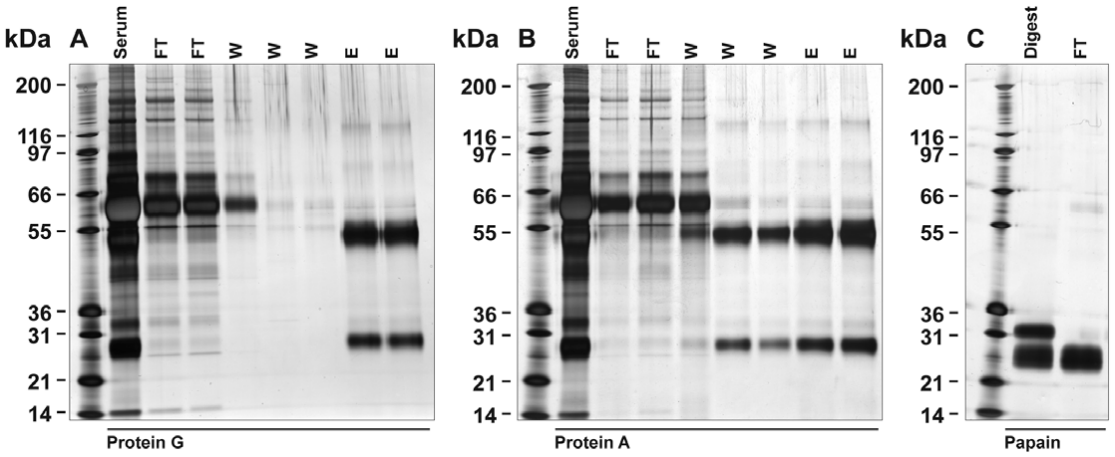
Purification of *P. alecto* IgG and generation of Fc and Fab fragments. Panel A; Protein G purification. Panel B; Protein A purification. Lane 1 (all panels); Mark 12 standard. FT; flow-through, W; wash, E; elution. Panel C; papain digestion containing Fab and Fc fragments was fractionated on immobilised Protein A column. Flow through (FT) fraction contained the Fab fragment (a doublet of approximately 25 kDa).

Commercially available immobilised Proteins G and A successfully captured whole IgG from *P. alecto* serum and plasma. It appeared that *P. alecto* IgG had greater binding affinity to Protein G than to Protein A (Fig. 2A and B). Washing removed significantly more IgG from the immobilised Protein A column compared to Protein G. IgG binding to Protein L was negligible (data not shown). IgG_H_ was approximately 50–52 kDa. A light chain immunoglobulin of approximately 25 kDa was also visible in Protein G purified fractions (Fig. 2A). Papain fragmentation of Protein G purified IgG revealed that the *P. alecto* immunoglobulin was significantly more resistant to protease cleavage compared to rabbit IgG. The rabbit IgG was completely digested after 12 h incubation (data not shown) while almost complete fragmentation of *P. alecto* IgG could only be achieved after 18 h incubation with 5-fold higher enzyme concentration (Fig. 2C). Following digestion, the Fab and Fc fragments were separated by Protein A affinity chromatography. The Fc (Fcγ) fragment was bound to the column and later eluted, whilst the Fab fragment did not bind and was collected in the flow through fraction. This method isolated approximately 5.4 mg Fab from 1 ml of serum. Purified whole IgG, IgG_H_, Fab fragments and Fcγ fragments were used as antigens for production of specific polyclonal antibodies in rabbits.

#### IgM enrichment by anti-Fab affinity chromatography

**Figure 3:**
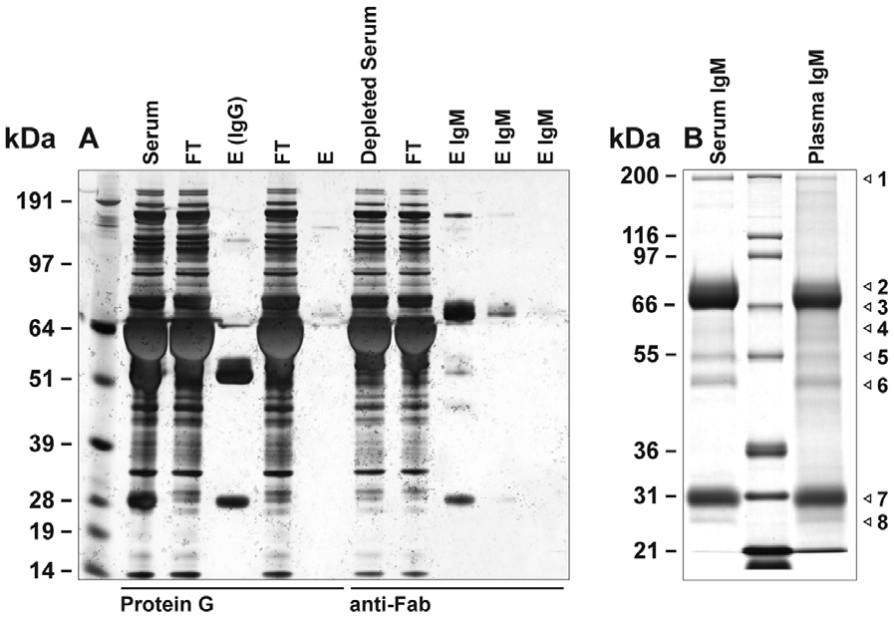
Purification of *P. alecto* IgM from IgG depleted serum on immobilised anti-Fab-specific antibody. Panel A; purification of IgM from IgG depleted serum. Immobilised Protein G column was used to deplete IgG from whole serum. The immobilised anti-Fab-specific antibody column was then used to purify IgM. Lane 1; See Blue plus 2 markers. FT; flow-through, E; elution. Panel B; purified IgM fractions from serum (lane 1) and plasma (lane 3). Lane 2; Mark 12 standard. Selected protein bands (labelled 1 to 8) were excised from gels for LC-MS/MS analysis.

Immobilised anti-Fab-specific antibody was used to capture non-IgG immunoglobulins (IgM and IgA) from IgG-depleted *P. alecto* serum and plasma samples. Two major bands were detected by reducing SDS-PAGE in the eluate from both serum and plasma samples; a 66–70 kDa band representative of IgM_H_, and a 25 kDa band representative of immunoglobulin light chain (Fig. 3A and 3B). No band representative of IgA_H_ (predicted 52–55 kDa from mRNA sequence [28]) was observed. To confirm the identities of these bands and others, the putative IgM_H_ band, its associated immunoglobulin light chain, and other minor bands (highlighted by arrows, Fig. 3B lanes 1 and 3) were excised and subjected to LC-MS/MS (Table 1). Bands 1 to 6 in serum and plasma contained protein corresponding only to Cμ, immunoglobulin light chain and/or J chain. However, band 4 in serum samples also contained sequence corresponding to Cα. A similar result was obtained when the IgM/IgA enriched fractions from serum and plasma were pooled and subjected to SEC (Fig. S1A and S1B). Four fractions from each serum and plasma were analysed by reducing SDS-PAGE and LC-MS/MS. All fractions from plasma were found to correspond to Cμ, immunoglobulin light chain and/or J chain. One fraction from serum was also shown to correspond to Cα (Table S1). Gel eluted IgM_H_ was used as antigen for production of specific polyclonal antibodies in rabbits.

**Table 1:** LC-MS/MS analysis of major gel bands from Fab-purified *P. alecto*.

Gel band number	MWt (kDa)	Protein identity	Peptides found	Sequence coverage (%)
MS1	200	IgM_H_ (Cμ)	13	19.6
MS2	83	IgM_H_ (Cμ)	15	16.4
MS3	74	IgM_H_ (Cμ)	17	19.5
MS4	61	IgM_H_ (Cμ)	6	16.8
		IgA_H_ (Cα)	8	21.3
MS5	54	IgM_H_ (Cμ)	4	11.0
MS6	48	IgM_H_ (Cμ)	2	5.4
MS7	30	Light chain	6	30.6
MS8	28	Light chain	3	23.0
		J chain	3	17.7
MP1	200	IgM_H_ (Cμ)	3	7.0
MP2	83	IgM_H_ (Cμ)	10	14.6
MP3	74	IgM_H_ (Cμ)	11	15.1
MP4	61	IgM_H_ (Cμ)	5	5.6
MP5	54	IgM_H_ (Cμ)	8	11.8
MP6	48	IgM_H_ (Cμ)	2	5.4
MP7	30	Light chain	5	18.7
MP8	28	Light chain	2	15.3
		J chain	4	26.6

Serum bands 1–8 from Figure 3B are denoted MS1 to MS8, plasma bands 1–8 from Figure 3B are denoted MP1 to MP8.

### Characterisation of *P. alecto* immunoglobulins

#### Distribution and expression

**Figure 4:**
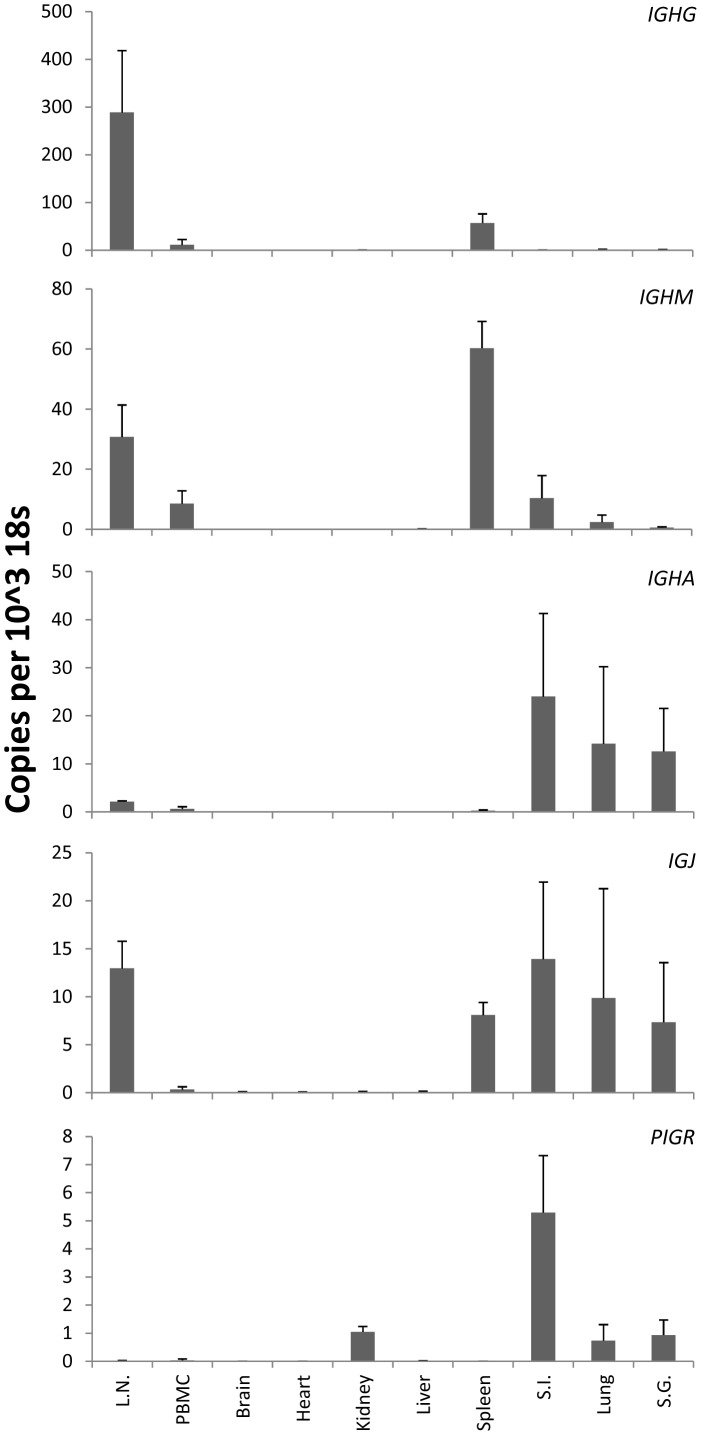
Quantitative measurement of *IGHG*, *IGHM*, *IGHA*, *IGJ* and *PIGR* mRNA in *P. alecto* tissues. mRNA transcripts were measured by SYBR Green qPCR and normalised to 18 s ribosomal RNA. Results show the mean ± standard deviation of n = 3 individual healthy wild-caught bats. Abbreviations: L.N., lymph node; S.I., small intestine; S.G., salivary gland; PBMC, peripheral blood mononuclear cells.

The distribution of IgG, IgM and IgA in healthy *P. alecto* was examined using LC-MS/MS analysis of gel-separated proteins from various tissue extracts and secretions (Fig. S2). No proteins of high molecular weight were detected in faeces (data not shown) and these samples were excluded from further analysis. IgG_H_ was detected in all samples with the exception of tears. In contrast, IgM_H_ was only detected in lung lavage while IgA_H_ was identified in the small and large intestine lavages, milk and tears (Table 2). Due to the apparent low abundance of IgA in tissues and secretions where it may be expected to be found, we examined the *IGHG*, *IGHA*, *IGHM*, *IGJ* and *PIGR* mRNA expression levels in ten tissues collected from three individual wild-caught *P. alecto* fruit bats by qPCR (Fig. 4). *IGHA* mRNA was highly transcribed in the small intestine, lung and salivary gland. *IGHM* was highly expressed in lymph node and spleen, and moderately transcribed in peripheral blood mononuclear cells (PBMC), small intestine and lung. *IGJ and PIGR* were strongly expressed in small intestine, lung and salivary gland. Brain, heart, kidney and liver expressed very low levels of all three mRNAs. *IGHG* was most abundant in the lymph node, spleen and PBMC.

**Table 2:** Detection of immunoglobulin heavy chains by LC-MS/MS of gel-purified tissue lavages, extracts and secretions.

	IgM_H_		IgG_H_		IgA_H_	
Sample	Peptides found	Sequence coverage (%)	Peptides found	Sequence coverage (%)	Peptides found	Sequence coverage (%)
Lung lavage	4	16.0	10	33.2	-	-
Small intestine lavage	-	-	9	30.4	3	8.9
Large intestine lavage	-	-	8	23.1	8	17.2
Small intestine extract	-	-	3	9.9	-	-
Large intestine extract	-	-	3	8.6	-	-
Salivary gland extract	-	-	7	20.0	-	-
Milk	-	-	8	24.4	4	20.0
Tears	-	-	-	-	11	34.7

Samples were run on separate gels and bands were excised from the region predicted to comprise the immunoglobulin heavy chains (50–80 kDa). Gel images are presented in Figure S2.

#### Glycosylation

**Figure 5:**
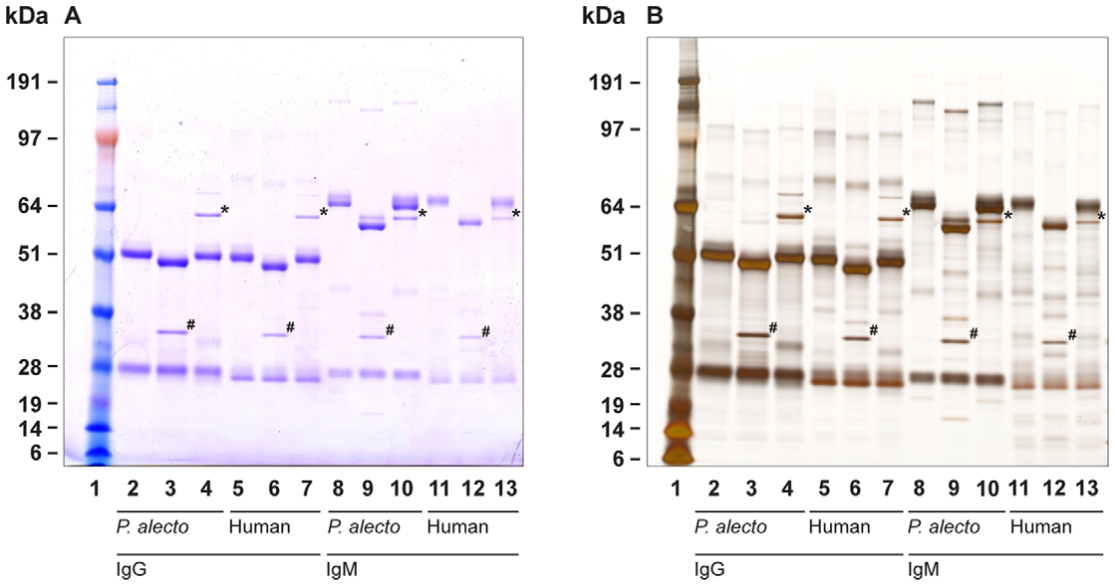
Electrophoretic characterisation of *P. alecto* and human IgG and IgM before/after deglycosylation. Proteins were visualised with Coomassie blue (panel A) or silver nitrate (panel B). Bands with asterisks indicate neuraminidase and hashes indicate PNGaseF used for deglycosylation. Lane 1, See Blue plus 2 markers; lanes 2–4, *P. alecto* IgG; lanes 5–7, human IgG; lanes 8–10, *P. alecto* IgM; lanes 11–13, human IgM; lanes 3, 6, 9 and 12, PNGaseF treatment; lanes 4, 7, 10 and 13, neuraminidase treatment.

The glycosylation status of whole *P. alecto* IgG and IgM was assessed by treatment with glycosidases and lectin binding. Human immunoglobulins were used as controls. Electrophoretic analysis of immunoglobulin subunits following deglycosylation revealed greater glycan content associated with IgM molecules than IgG which was observed as a greater mass differential following deglycosylation (Fig. 5A and 5B). This observation was confirmed by staining of IgG and IgM with a glycan specific stain (data not shown). Following deglycosylation with PNGaseF, two bands of IgM_H_ were observed in *P. alecto*. It can be estimated from the mass differences that bat IgG_H_ and IgM_H_ have approximately 1–2 and 3–4 occupied glycosylation sites, respectively.

Glycan composition was analysed through lectin binding and revealed that the overall structure of IgG and IgM glycans was most likely *N*-linked di-antenary. Reactivity with DSA and GNA indicated the presence of 1,4-GlcNAc and 1,3-mannose component residues (Fig. S3) and the reactivity with MAA II and SNA indicated the presence of sialic acid residue(s), most likely in an α2→3 configuration (Fig. S3C and S3D). Treatment with PNA and UEA I indicated the presence of galactose and fucose residues; however, these tests were less conclusive, possibly due to incomplete deglycosylation or nonspecific binding of the lectins.

#### Diversity of IgG_H_ and IgM_H_ isoforms

**Figure 6:**
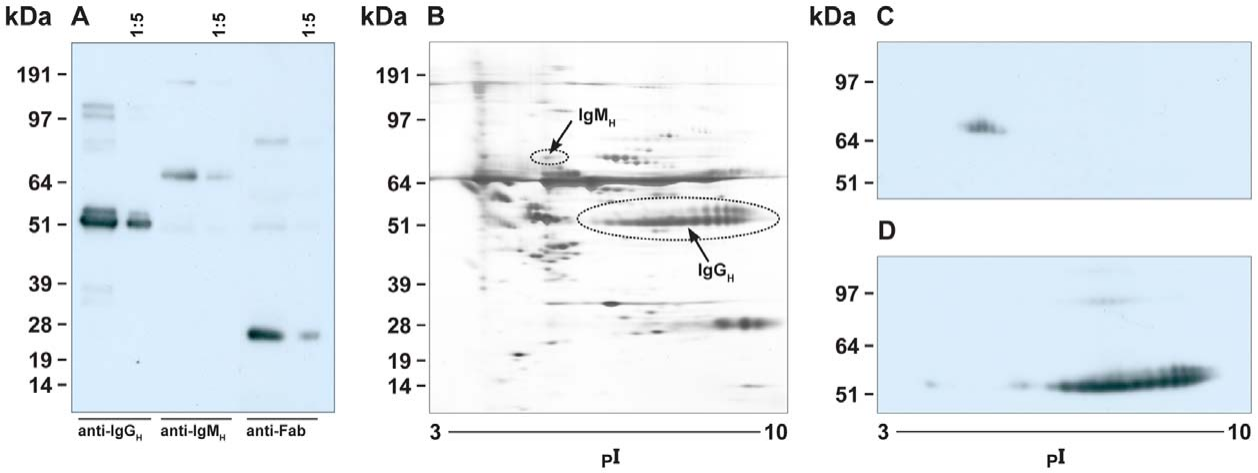
Reactivity of rabbit antibodies to *P. alecto* IgG_H_ and IgM_H_ in *P. alecto* serum. Panel A. *P. alecto* serum samples (neat and 1∶5) were separated by reducing SDS-PAGE and probed with rabbit antiserum against *P. alecto* IgG_H_ (lanes 1 and 2), IgM_H_ (lanes 3 and 4) and Fab fragment (lanes 5 and 6). Panel B. 2-DE separation of *P. alecto* serum sample (silver stain). Panel C and D, 2-DE separation of *P. alecto* serum sample probed with rabbit antiserum against *P. alecto* IgM_H_ (panel C) and IgG_H_ (panel D).

Antiserum generated against IgG_H_ reacted predominantly with the two bands representative of IgG_H_ in *P. alecto* serum (Fig. 6A). The antiserum generated against IgM_H_ reacted predominantly with a single band representative of IgM_H_ in *P. alecto* serum (Fig. 6A). The anti-Fab antiserum reacted only with immunoglobulin light chains (Fig. 6A). Immunoblotting of 2-DE separated *P. alecto* serum revealed the presence of a diverse range of IgG_H_ and IgM_H_ isoforms (Fig. 6B, 6C and 6D). IgG_H_ antiserum reacted with a variety of IgG_H_ isoforms ranging in pI from 5–9 (Fig. 6D). In contrast, the IgM_H_ antiserum reacted with a less complex IgM_H_ repertoire (Fig. 6C).

#### Reactivity of IgG_H_ and IgM_H_ antiserum

**Figure 7:**
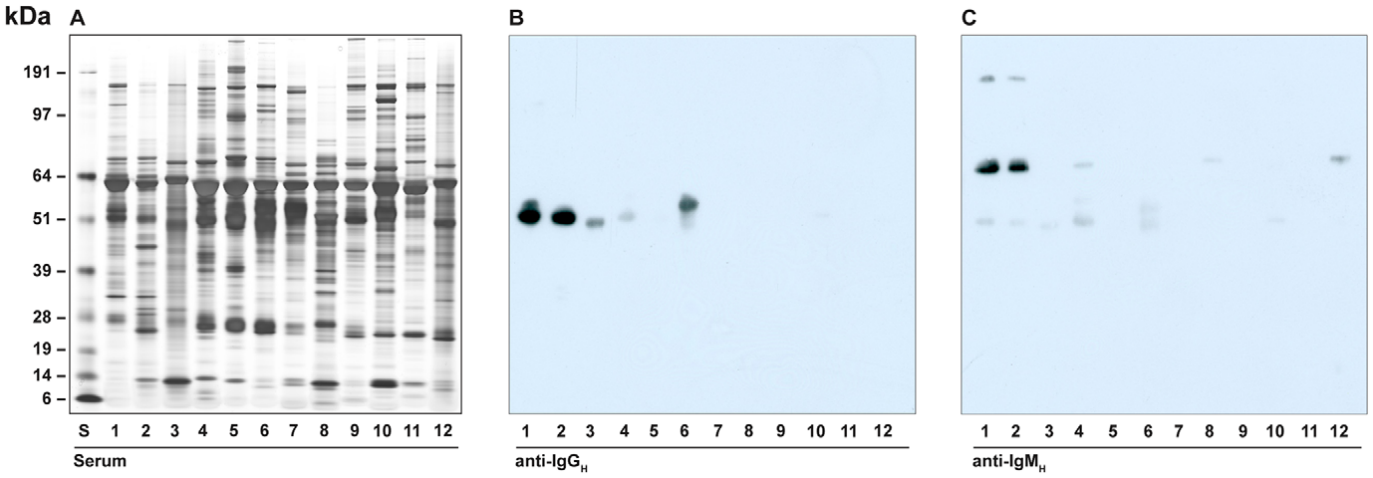
Cross-species reactivity of rabbit anti-*P. alecto* IgG_H_ and IgM_H_. Panel A; Silver stained gel of serum samples from different animals. Lanes 1; *P. alecto*; 2, *P. conspiculatus*; 3, *R. megaphylus*; 4, human; 5, Tasmanian devil; 6, horse; 7, cow; 8, cat; 9, pig; 10, mouse; 11, chicken; 12, ferret. S, See Blue plus 2 markers. Panel B; western blot of serum samples from panel A using *P. alecto* anti-IgG_H_ (lanes same as panel A). Panel C; Immunoblot of serum samples from panel A using *P. alecto* anti-IgM_H_ (lanes same as panel A).

Serum proteins for a number of species were separated by reducing SDS-PAGE (Fig. 7A). Antiserum generated against *P. alecto* IgG_H_ showed reactivity to IgG_H_ of the fruit bats *P. conspicillatus* and, to a lesser extent, *Rosettus megaphylus* (Fig. 7B, lanes 2 and 3 respectively). Reactivity was also detected with horse IgG_H_ (lane 6). Antiserum generated against *P. alecto* IgM_H_ reacted with *P. conspicillatus* and very weakly with ferret IgM_H_ (Fig. 7C).

### Attempted purification of IgA

Consistent with previous reports [39]–[41], human IgA was purified from IgG-depleted human serum and plasma using immobilised ligands (Jacalin, Peptide M and SSL7) (data not shown). Immobilised-Peptide M and SSL7 bound no protein from *P. alecto* plasma and serum, and immobilised Jacalin bound approximately 19 bands with varying molecular masses (Fig. S4A, S4B, S4C). Protein fractions eluted from the Jacalin column were analysed by reducing SDS-PAGE and 19 distinct protein bands were tested for the presence of IgA by LC-MS/MS (Fig. S4D). None of these proteins contained peptides representative of IgA (Table S2)

## Discussion

IgA is the second most abundant immunoglobulin class in normal human serum, accounting for approximately 15–20% of the total immunoglobulin [20]. The immobilised anti-Fab-specific antibody used within this study clearly enriched IgM from IgG depleted *P. alecto* serum. However, no visible enrichment of IgA was observed. Trace quantities of serum IgA were detected only by LC-MS/MS. Parallel experiments with anti-human Fab antibody successfully enriched IgA from human serum,. Furthermore, affinity chromatography with ligands known to be specific to IgA [39]–[41] failed to enrich this immunoglobulin from *P. alecto* serum. It must be kept in mind the approaches utilised in this study to isolate IgA are based on the assumption that *P. alecto* IgA would have similar characteristics to mammalian IgA and the variable heavy chain regions would be shared between the major immunoglobulin classes. Previous mRNA sequencing studies of the *P. alecto* immunoglobulin repertoire would suggest this assumption is sound [28]. However, if *P. alecto* contains additional atypical IgA-like molecules, our approaches may have failed to detect such variants. Various IgA-like equivalents have been identified across a range of species including teleost fish, amphibians and reptile [48]–[50], highlighting the divergent evolutionary pathways that contribute to mucosal immunity.

Previous studies on a range of bat species found that the bat IgA gene sequence more closely resembles human IgA2. This similarity is due, in part, to the absence of a cysteine in the constant heavy 1 domain that is part of the covalent disulfide bond to the light chain [29]. The IgA_H_ sequence previously obtained for *P. alecto* is also missing this cysteine [28]. In humans, IgA2 is the dominant IgA subclass in mucosal secretions, whereas IgA1 is more common in serum. If bats contain only a single IgA2-like class, we may expect to find a higher concentration of IgA in mucosal secretions compared to that found in serum.

The apparent scarcity of serum IgA led us to further investigate the abundance of IgA in various mucosal secretions and tissues. LC-MS/MS analysis detected IgA in the small and large intestine lavages, milk and tears. The absence of IgA in the lung lavage and salivary gland may be the result of differential regulation of either IgA itself or the J-chain or pIgR. Real-time PCR revealed both the *IGJ* and *PIGR* transcripts co-localised with *IGHA* across most tissues. *PIGR* and *IGJ* were most abundantly expressed in the small intestine where both IgA transcript and protein was detected. In contrast the expression of *PIGR* was markedly lower in the lung and salivary gland which may have contributed to the lower abundance of IgA protein in these tissues.

As expected *IGHG* was the most highly expressed immunoglobulin mRNA in the lymph node, spleen and PBMC. Interestingly, while *IGHG* mRNA was not highly expressed in the mucosal tissues, LC-MS/MS revealed IgG was the dominant immunoglobulin in these tissues and secretions. This result suggests the majority of IgG protein within the mucosal tissues and secretions is not locally produced and is more likely transported to these sites. In humans, the transportation and regulation of IgG across the mucosal epithelium is driven by the neonatal Fc receptor [51]. The importance of mucosal IgG in the defence against epithelium associated pathogens has been demonstrated previously and is now recognised as playing a key role in mucosal immunity along with IgA [52]. The relative abundance of IgG over IgA within the milk of *P. alecto* may have implications for the transfer of material immunity. In humans the majority of IgG is transferred to the fetus via the placenta and only trace quantities of IgG are found in lacteal secretions [53], [54]. Other species, such as cows, sheep and horses do not transfer IgG across the placenta and the majority of IgG, and a smaller proportion of IgA, is provided to the newborn through the colostrum and milk [53], [54]. The data presented herein would suggest *P. alecto*, unlike humans and rabbits, transfers the majority of IgG through the lacteal secretions rather than through the placenta.

Selective IgA deficiency is a common condition in humans affecting approximately 1 in 600 individuals [55]. In most cases individuals deficient in IgA show no clinical symptoms. However, severe allergies, autoimmune and gastrointestinal disorders have been reported in some patients [55]. Studies in IgA knockout mice infected with influenza virus reported similar levels of pulmonary virus infection in IgA^−/−^ and IgA^+/+^ mice, suggesting IgA is not required for the prevention of disease [56]. Interestingly, however, the IgA deficient mice produced higher quantities of systemic IgG. Similar findings have also been reported in humans [57], [58]. Indeed, significantly higher levels of IgM and IgG were found in the saliva of IgA deficient individuals compared to normal healthy controls [58]. In light of these studies, we hypothesise that the dominance of IgG in the mucosal secretions of *P. alecto* may be an evolutionary compensation for an inherent lack of IgA. Further research is required to determine what functional relevance this would have on the immune response.

2-DE resolution of *P. alecto* serum demonstrated the existence of multiple IgG_H_ isoforms. The immunoblot reactive spot train spanned a wide pI range of 5 to 9. This range is in agreement with the predicted pI range of 15 separate *IGHG* mRNA previously sequenced (range of 6.3 to 8.3) [28]. A doublet of slightly different molecular weights for the IgG_H_ chain was also observed after 1-DE immunoblotting. Most mammalian species contain multiple IgG subclasses and therefore it is not surprising that *P. alecto* contained multiple subclasses of IgG. Indeed, Butler *et al.* [29] reported the presence of multiple *IGHG* subclass transcripts in a number of bat species including *Myotis lucifugus*, *Eptiscus fuscus* and *Cynopterus sphinx*. In contrast to IgG_H_, a much smaller number of reactive IgM_H_ isoforms were detected in serum following 2-DE western blotting. However, following deglycosylation with PNGaseF, two bands of IgM_H_ were observed in *P. alecto*. This result was not observed in human IgM. This finding suggests the possible existence of a subclass of IgM_H_ in *P. alecto* that is absent from humans. To our knowledge the existence of multiple IgM subclasses has not been previously described in bats. Considering that IgM provides a first line of defence against microbial invasion, diversification of this immunoglobulin class may confer a distinct immunological advantage to the species. Indeed, different IgM subclasses may confer an enhanced and diverse classical complement pathway which, to some extent, may compensate for the lack of serum IgA in *P. alecto*. Further studies are required to elucidate the functional significance of putative IgM subclasses within *P. alecto*.

*P. alecto* IgG from serum and plasma binds to both Protein A and Protein G but has significantly greater binding affinity to Protein G. Protein G recognises most IgG subclasses in many species [59] whilst Protein A binds only certain IgG subclasses and in some species does not bind IgG (e.g. rat and goat). This finding may reflect the presence of different *P. alecto* IgG subclasses with different binding affinity to Protein A. *P. alecto* IgG_H_ antiserum cross reacted strongly against two other bats (*P. conspiculatus* and *R. megaphylus*) and horse IgG_H_. A close evolutionary relationship between chiroptera bats and horse has been reported on a number of occasions and may have contributed to this cross reactivity [60], [61]. The fact that *P. alecto* IgM_H_ antiserum did not cross react with horse suggests this immunoglobulin class has diverged more rapidly since the separation of horse and bats compared to IgG. Divergence of IgM may have contributed to the rise of different IgM subclasses as described above. Given that serum IgA is drastically reduced in *P. alecto*, diversification of IgM may have functional significance for the early antibody response.

The novel method involving the use of immobilised anti-Fab-specific antibody described herein allows for the simultaneous purification of immunoglobulin classes from serum and production of class-specific antiserum without prior knowledge of the species' immunoglobulin repertoire. IgG and IgM were purified from *P. alecto* serum, and rabbit antiserum specific to both molecules were produced. While IgG and IgM were successfully isolated from serum, no visible enrichment of serum IgA was achieved. Trace quantities of IgA were detected only by mass spectrometry in serum. IgA was also detected in the mucosal secretions of the small and large intestine lavages, milk and tears. The glycosylation pattern of *P. alecto* IgG and IgM appears typical of that described for other mammalian immunoglobulins. Furthermore deglycosylation of IgM_H_ and 2-DE immunoblotting of IgG_H_ revealed the possible existence of multiple IgM_H_ and IgG_H_ subclasses in *P. alecto*. Taken together, this study suggests healthy *P. alecto* bats have considerably less serum IgA than expected. Diversification of IgM and IgG subclasses may be an evolutionary compensation for this lack of IgA. Further research is now required to investigate the functional relevance of this unique antibody repertoire, particularly in response to viral infection. The knowledge and tools developed here will facilitate this research.

## Supporting Information

Figure S1 **Separation of affinity purified IgM by SEC.** Panel A, example of separation for IgM from plasma (separation of affinity purified IgM from serum was similar; data not shown). Panel B, reducing SDS-PAGE analysis of numbered fractions, lane 1, See Blue plus 2 markers; lane 2, plasma sample; lane 3, Fab purified IgM; lane 4, SEC fraction 1; lane 5, SEC fraction 2; lane 6, SEC fraction 2 diluted 1∶5; lane 7, SEC fraction 3; lane 8, SEC fraction 4.
(TIF)

Figure S2 **SDS-PAGE fractionation of proteins derived from tissue lavages, extracts and secretions visualised with Coomassie blue.** Each sample was run in triplicate in order to obtain sufficient material for LC-MS/MS. Arrow heads indicate distinct bands or regions that were excised from gels for MS analysis. Arrow heads *red*, *green*, *yellow* and *blue* represent regions were peptides corresponding to IgG_H_, IgM_H_, IgA_H_ and pIgR were obtained respectively. Each gel contains a See Blue plus 2 marker.
(TIF)

Figure S3 **Electrophoretic mobility, heterogeneity and lectin affinities of** ***P. alecto*** **and human IgG and IgM.** Proteins were visualised following probing with lectins: A, PNA; B, GNA; C, UEA I; D, MAA II; E, SNA; F, DSA. Lane 1, molecular mass markers; lanes 2–4, *P. alecto* IgG; lanes 5–7, human IgG; lanes 8–10, *P. alecto* IgM; lanes 11–13, human IgM; lanes 3, 6, 9 and 12, PNGaseF treatment; lanes 4, 7, 10 and 13, neuraminidase treatment.
(TIF)

Figure S4 **Attempted purification of IgA by affinity chromatography.** Peptide M (Panel A), Peptide SSL7 (Panel B) and Jacalin (Panel C). Panel D. Eluted *P. alecto* proteins from immobilised Jacalin by affinity chromatography. Bands 1–19 were excised and subjected to LC-MS/MS (Table S2)
(TIF)

Table S1 **Protein identification by LC-MS/MS analysis of IgM affinity purified fractions.** The four fractions were separated by SEC from *P. alecto* serum and plasma (see Figure S1).
(DOCX)

Table S2 **Protein identification by LC-MS/MS analysis of 19 excised bands.** Proteins were purified by Jacalin affinity chromatography from IgG depleted *P. alecto* serum (see Figure S3D).
(DOCX)
