# Supplementary figures and images for: Purification and Characterisation of Immunoglobulins from the Australian Black Flying Fox (Pteropus alecto) Using Anti-Fab Affinity Chromatography Reveals the Low Abundance of IgA
Source: PLoS One. 2013 Jan 7;8(1):e52930. doi: 10.1371/journal.pone.0052930 (PMC3538733; doi:10.1371/journal.pone.0052930)

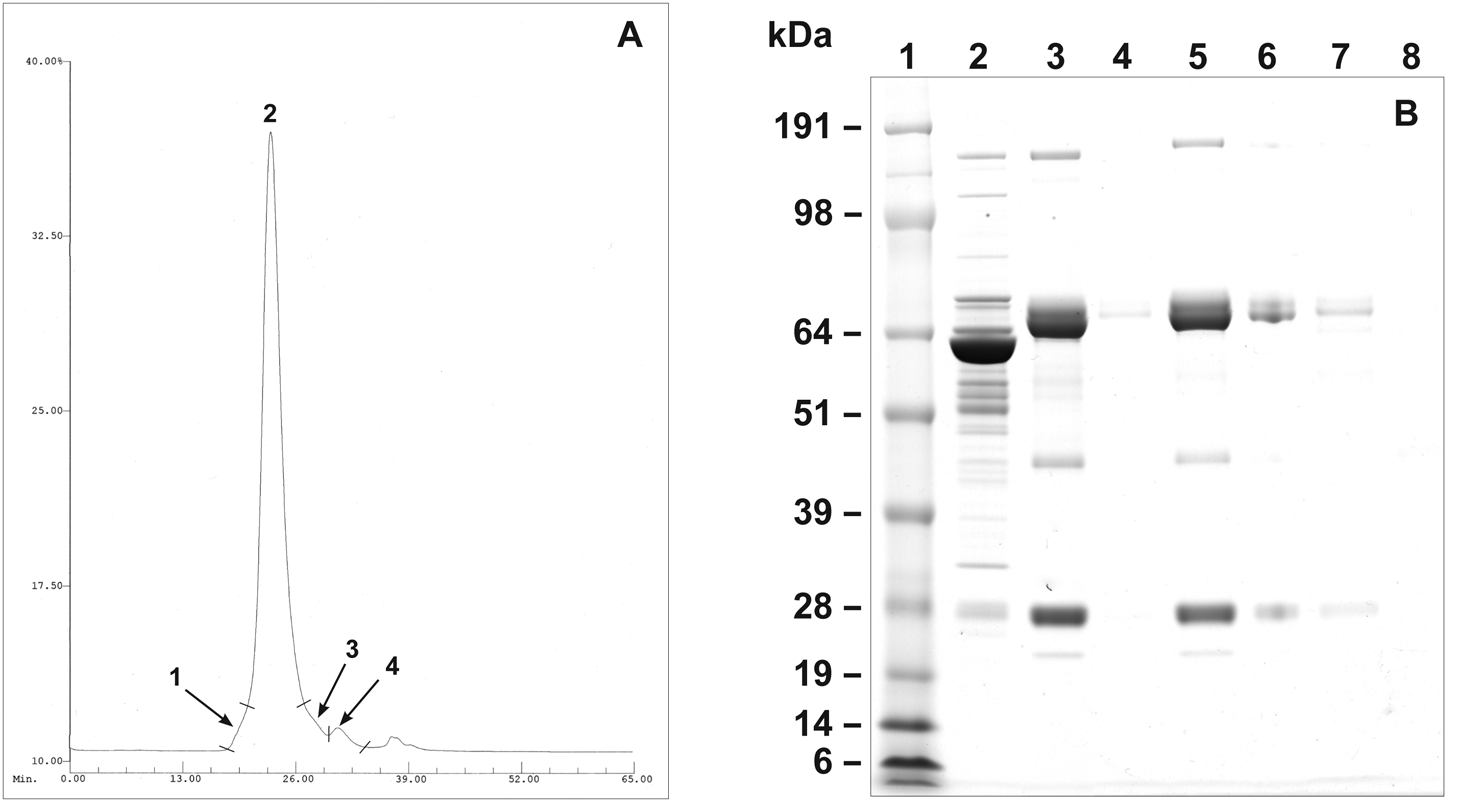

Supplement: Figure S1 — Separation of affinity purified IgM by SEC. Panel A, example of separation for IgM from plasma (separation of affinity purified IgM from serum was similar; data not shown). Panel B, reducing SDS-PAGE analysis of numbered fractions, lane 1, See Blue plus 2 markers; lane 2, plasma sample; lane 3, Fab purified IgM; lane 4, SEC fraction 1; lane 5, SEC fraction 2; lane 6, SEC fraction 2 diluted 1∶5; lane 7, SEC fraction 3; lane 8, SEC fraction 4. (TIF) [file pone.0052930.s001.tif]

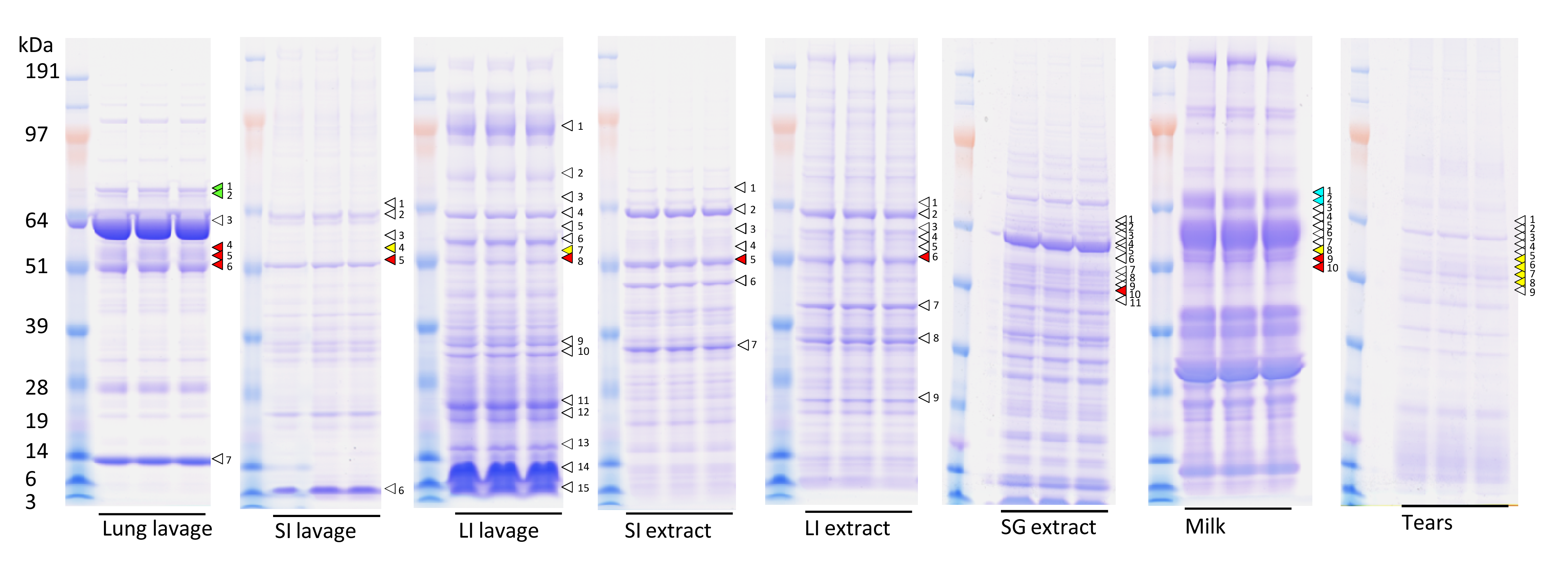

Supplement: Figure S2 — SDS-PAGE fractionation of proteins derived from tissue lavages, extracts and secretions visualised with Coomassie blue. Each sample was run in triplicate in order to obtain sufficient material for LC-MS/MS. Arrow heads indicate distinct bands or regions that were excised from gels for MS analysis. Arrow heads red, green, yellow and blue represent regions were peptides corresponding to IgGH, IgMH, IgAH and pIgR were obtained respectively. Each gel contains a See Blue plus 2 marker. (TIF) [file pone.0052930.s002.tif]

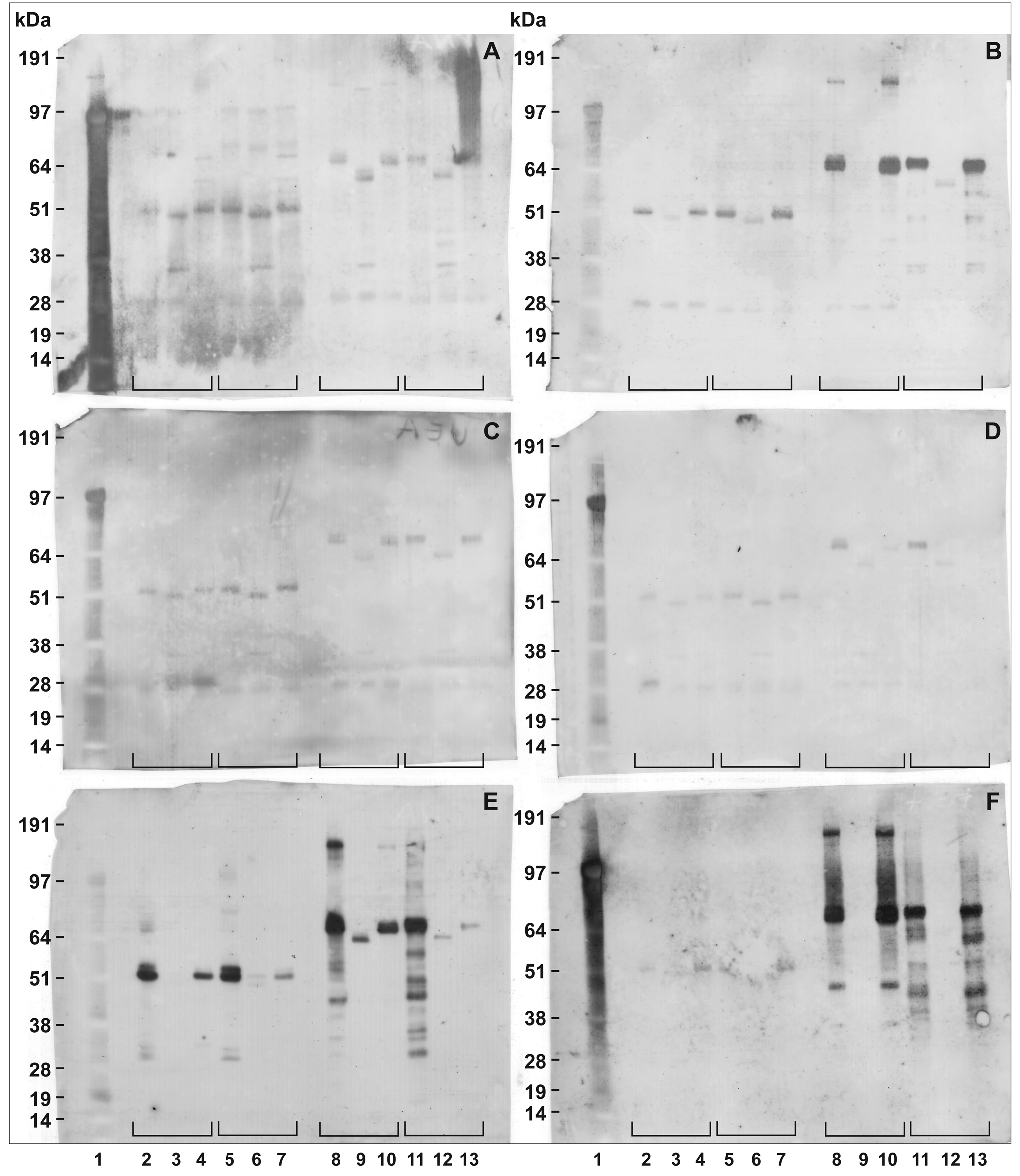

Supplement: Figure S3 — Electrophoretic mobility, heterogeneity and lectin affinities of P. alecto and human IgG and IgM. Proteins were visualised following probing with lectins: A, PNA; B, GNA; C, UEA I; D, MAA II; E, SNA; F, DSA. Lane 1, molecular mass markers; lanes 2–4, P. alecto IgG; lanes 5–7, human IgG; lanes 8–10, P. alecto IgM; lanes 11–13, human IgM; lanes 3, 6, 9 and 12, PNGaseF treatment; lanes 4, 7, 10 and 13, neuraminidase treatment. (TIF) [file pone.0052930.s003.tif]

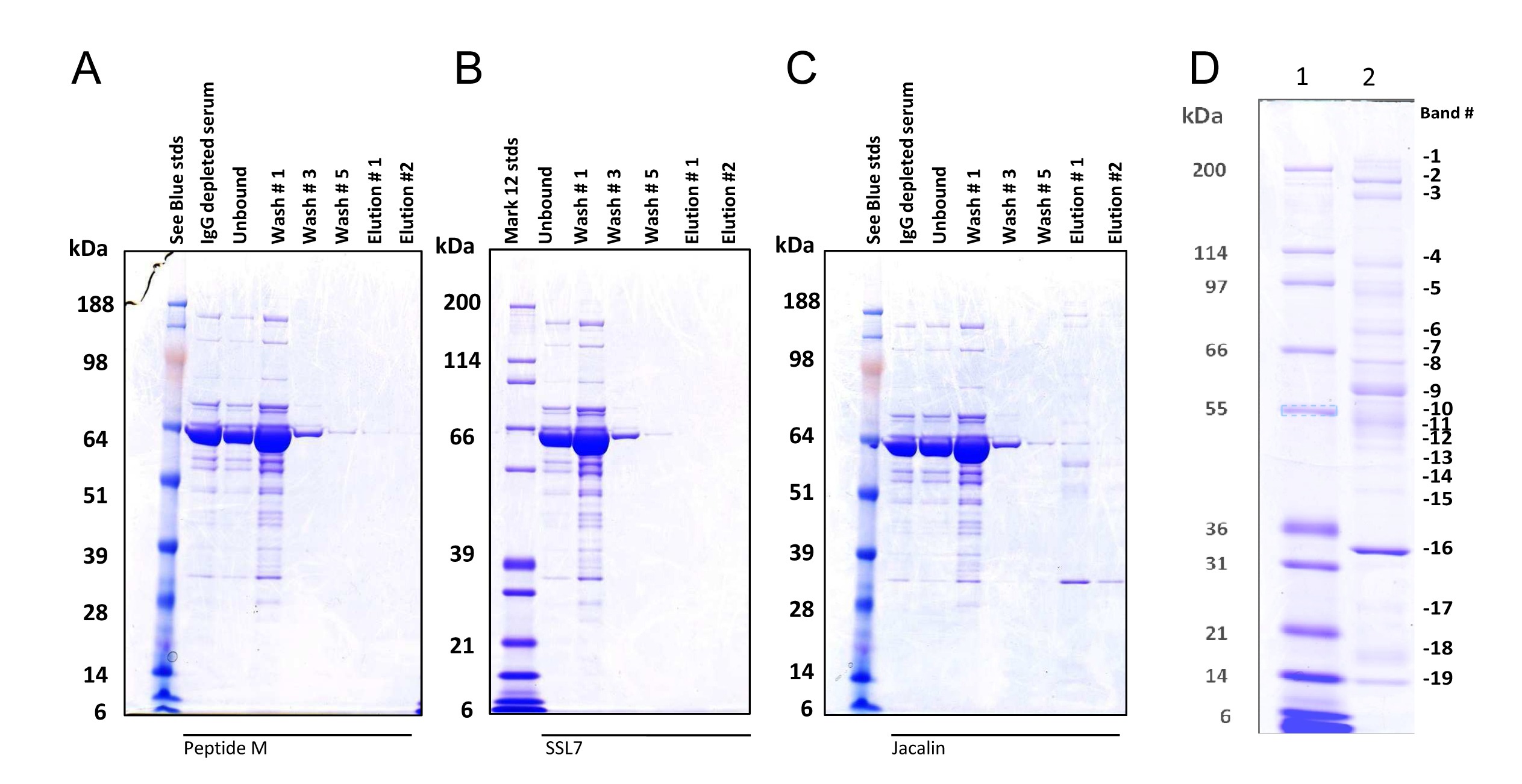

Supplement: Figure S4 — Attempted purification of IgA by affinity chromatography. Peptide M (Panel A), Peptide SSL7 (Panel B) and Jacalin (Panel C). Panel D. Eluted P. alecto proteins from immobilised Jacalin by affinity chromatography. Bands 1–19 were excised and subjected to LC-MS/MS (Table S2) (TIF) [file pone.0052930.s004.tif]
