# Supplementary material for: Purification and Characterisation of Immunoglobulins from the Australian Black Flying Fox (Pteropus alecto) Using Anti-Fab Affinity Chromatography Reveals the Low Abundance of IgA
Source: PLoS One. 2013 Jan 7;8(1):e52930. doi: 10.1371/journal.pone.0052930 (PMC3538733; doi:10.1371/journal.pone.0052930)
Supplement: Table S1 — Protein identification by LC-MS/MS analysis of IgM affinity purified fractions. The four fractions were separated by SEC from P. alecto serum and plasma (see Figure S1). (DOCX) [file pone.0052930.s005.docx]

**Table S1.**

| Fraction # | Type | Proteins identified | Peptides found | Coverage (%) |
| --- | --- | --- | --- | --- |
| SV1 | Serum | Nil | - | - |
| SV2 | Serum | IgM_H_ (Cµ) | 14 | 26.9 |
|  |  | Light chain | 3 | 23.0 |
|  |  | J chain | 3 | 22.2 |
| SV3 | Serum | IgM_H_ (Cµ) | 9 | 17.6 |
|  |  | IgA_H_ (Cα) | 4 | 10.3 |
|  |  | Light chain | 3 | 23.0 |
| SV4 | Serum | IgM_H_ (Cµ) | 5 | 12.1 |
|  |  | Light chain | 4 | 30.6 |
| PV1 | Plasma | Nil | - | - |
| PV2 | Plasma | IgM_H_ (Cµ) | 14 | 25.1 |
|  |  | Light chain | 4 | 23.0 |
|  |  | J chain | 2 | 17.1 |
| PV3 | Plasma | IgM_H_ (Cµ) | 3 | 9.1 |
| PV4 | Plasma | IgM_H_ (Cµ) | 2 | 5.4 |
|  |  | Light chain | 4 | 30.6 |
|  |  |  |  |  |
