# Supplementary material for: Purification and Characterisation of Immunoglobulins from the Australian Black Flying Fox (Pteropus alecto) Using Anti-Fab Affinity Chromatography Reveals the Low Abundance of IgA
Source: PLoS One. 2013 Jan 7;8(1):e52930. doi: 10.1371/journal.pone.0052930 (PMC3538733; doi:10.1371/journal.pone.0052930)
Supplement: Table S2 — Protein identification by LC-MS/MS analysis of 19 excised bands. Proteins were purified by Jacalin affinity chromatography from IgG depleted P. alecto serum (see Figure S3D). (DOCX) [file pone.0052930.s006.docx]

| **Band** | **Peptides found** | **Coverage %** | **Protein ID blastp vs NCBI nr** |
| --- | --- | --- | --- |
| 1 | 9 | 5.64 | Fibronectin-like isoform 1 |
| 2 | 6 | 14.85 | Inter-alpha globulin inhibitor H2 polypeptideInter-alpha |
|  | 6 | 9.38 | Trypsin inhibitor heavy chain H1-like |
| 3 | 3 | 2.89 | Alpha-2-macroglobulin-P precursor |
| 4 | 3 | 7.19 | Inter-alpha globulin inhibitor H2 polypeptide |
|  | 2 | 4.93 | Inter-alpha-trypsin inhibitor heavy chain H1-like |
| 5 | 11 | 27.67 | Complement component 1 inhibitor |
| 6 | 4 | 7.54 | Inter-alpha globulin inhibitor H2 polypeptide |
| 7 | - | - | No peptides found |
| 8 | 7 | 8.24 | Alpha-fetoprotein-like (albumin) |
| 9 | 5 | 15.14 | Kininogen 1 |
| 10 | 4 | 11.96 | Alpha-2-HS-glycoprotein precursor (fetuin) |
|  | 4 | 17.52 | Fibrinogen beta chain isoform 1 preprotein |
|  | 4 | 11.08 | Kininogen 1 |
| 11 | 7 | 16.47 | Alpha-2-HS-glycoprotein precursor (fetuin) |
| 12 | 7 | 16.08 | Alpha-2-HS-glycoprotein precursor (fetuin) |
| 13 | 3 | 10.39 | Alpha-2-HS-glycoprotein precursor (fetuin) |
|  | 2 | 3.20 | Fibrinogen alpha precursor |
| 14 | 3 | 9.8 | Alpha-2-HS-glycoprotein precursor (fetuin) |
| 15 | 5 | 22.69 | Apolipoprotein A-IV |
|  | 2 | 7.65 | Alpha-2-HS-glycoprotein precursor (fetuin) |
| 16 | 9 | 5.69 | Haptoglobin precursor isoform 2 (beta) |
| 17 | - | - | No peptides found |
| 18 | 4 | 1.67 | Haptoglobin precursor isoform 2 (alpha) |
| 19 | 9 | 67.35 | Hemoglobin beta subunit |
|  | 4 | 21.77 | Hemoglobin alpha subunit |
|  | 2 | 2.16 | Haptoglobin precursor isoform 2 (alpha) |

**Table S2.**
